# Effect of astragalus injection on left ventricular remodeling in HFmrEF: a systematic review and meta-analysis

**DOI:** 10.3389/fcvm.2024.1374114

**Published:** 2024-08-06

**Authors:** Xu Han, Lumei Huang, Geng Li, Xinglang Mou, Caihong Cheng

**Affiliations:** ^1^ Department of Anorectal, Chongqing Changshou Traditional Chinese Medicine Hospital, Chongqing, China; ^2^ Department of Cardiology, Traditional Chinese Medicine Hospital Dianjiang Chongqing, Chongqing, China

**Keywords:** astragalus injection, heart failure with mildly reduced ejection fraction, left ventricular remodeling, randomized controlled trials, systematic review, meta-analysis

## Abstract

**Objectives:**

The aim of this meta-analysis is to evaluate the effect of astragalus injection (AI) on left ventricular remodeling (LVR) in patients with heart failure with mildly reduced ejection fraction (HFmrEF).

**Methods:**

The randomized controlled trials (RCTs) of AI in treating HFmrEF were retrieved from 8 major English and Chinese electronic databases, up until November 30, 2023. To evaluate the methodological quality of the included studies, the Cochrane bias risk tool and the Modified Jadad Scale were employed. Stata 17.0 software was utilized for statistical analysis, sensitivity analysis, and assessment of publication bias.

**Results:**

Ten RCTs with 995 patients (562 males and 433 females) were identified. Meta-analysis indicated that compared to conventional treatment (CT), AI significantly improved LVR, specifically increasing left ventricular ejection fraction (LVEF, MD = 4.56, 95% CI: 3.68–5.44, *p* < 0.00001), decreasing left ventricular end-diastolic volume (LVEDV, MD = −7.89, 95% CI: −11.13 to −4.64, *p* < 0.00001), left ventricular end-diastolic diameter (LVEDD, MD = −4.18, 95% CI: −5.79 to −2.56, *p* < 0.00001), left ventricular end-systolic volume (LVESV, MD = −8.11, 95% CI: −11.79 to −4.43, *p* < 0.00001), and left ventricular end-systolic diameter (LVESD, MD = −3.42, 95% CI: −4.90 to −1.93, *p* < 0.00001). AI also improved clinical efficacy (RR = 4.62, 95% CI: 3.11–6.88, *p* < 0.00001), reduced N-terminal pro-brain natriuretic peptide (NT-pro BNP, MD = −27.94, 95% CI: −43.3 to −12.36) level, without increasing the incidence of adverse reactions (RR = 1.60, 95% CI: 0.59–4.29, *p* = 0.35). Sensitivity analysis confirmed the reliability of the merged results, and Begg's and Egger's tests showed no significant publication bias.

**Conclusion:**

The systematic review and meta-analysis revealed that combining AI with CT improves LVR without increasing adverse events in HFmrEF patients. However, caution is needed in interpreting the results due to limited evidence. Future high-quality RCTs are needed to support these conclusions.

**Systematic Review Registration:**

https://www.crd.york.ac.uk/prospero/, PROSPERO [CRD42022347248].

## 1 Introduction

Heart failure (HF) is a global epidemic with increasing prevalence, affecting approximately 20 million patients worldwide (1). It is characterized by a poor prognosis and is the main cause of hospitalization for adults aged 65 and above, with a 1-year mortality rate of 10%–35% and a 5-year mortality rate greater than 50% (2). Among the various subtypes of HF, heart failure with mildly reduced ejection fraction (HFmrEF) is notable, accounting for 10%–20% of HF cases (3). HFmrEF is specifically identified by a left ventricular ejection fraction (LVEF) of 41%–49% and is closely associated with left ventricular remodeling (LVR), the pathophysiological core of HFmrEF, and a significant factor in its poor prognosis. LVR is marked by an increase in left ventricular volume and a decrease in contractile force (4, 5). Given these challenges, research targeting LVR, particularly in HFmrEF, is of paramount importance.

In recent years, with a deeper understanding of the pathophysiological mechanisms of HFmrEF, more studies have focused on treatment strategies targeting LVR. LVR refers to structural and functional changes in the left ventricle after chronic heart failure or myocardial infarction. These changes lead to decreased cardiac function, causing disease progression and poor outcomes in patients (6). Studies have shown that improving LVR can slow the progression of HF and improve patient prognosis (7). Traditional pharmacological treatments include beta-blockers, aldosterone antagonists, and angiotensin receptor-neprilysin inhibitors (ARNI). While these drugs have shown some efficacy in improving the prognosis of HFmrEF patients, significant limitations and inadequacies remain (8). For instance, beta-blockers can reduce cardiac load but may cause side effects such as fatigue and sexual dysfunction (9). Aldosterone antagonists are effective in reducing myocardial fibrosis but may lead to hyperkalemia (10). ARNIs show promise in improving prognosis, yet their long-term safety requires further investigation (11). Moreover, current treatments mainly focus on symptom control and do not fully address the fundamental issue of LVR (12). Therefore, finding new therapeutic methods that can effectively improve LVR has become a major research focus.

*Astragalus membranaceus*, a dried root of traditional Chinese medicine, has a long history of medicinal use for its various healing properties. It is renowned for its ability to nourish qi, promote blood circulation, unblock collaterals, induce diuresis, and reduce swelling (13). Astragalus injection (AI), derived from this root through water extraction and alcohol precipitation, contains active ingredients like flavonoids, saponins, polysaccharides, amino acids, and trace elements (14). These components endow AI with anti-inflammatory, antioxidant, and antiviral properties, and importantly, the potential to improve ventricular remodeling, a critical aspect of HFmrEF (13). Animal studies have shown that AI can mitigate LVR and cardiac damage in specific models, and a meta-analysis has revealed its benefits in immune regulation and inflammation reduction, particularly in viral myocarditis patients (15, 16). However, the specific impact of AI on LVR in HFmrEF patients remains an under-researched area.

To bridge this gap, our study aims to conduct a comprehensive meta-analysis of clinical randomized controlled trials (RCTs) focusing on the effects of AI on LVR in HFmrEF patients. This endeavor seeks to objectively evaluate AI's therapeutic potential in this context, thereby providing valuable insights for clinical application and guiding future research in managing HFmrEF.

## 2 Materials and methods

### 2.1 Study registration

This study adheres to established guidelines and maintains transparency through registration in the PROSPERO database (CRD42022347248). Consistent with the PRISMA guidelines, our report follows these rigorous standards for systematic reviews and meta-analysis (17).

### 2.2 Search strategy

A comprehensive search across eight major databases, including PubMed, Embase, Web of Science, Cochrane Library, China Knowledge Infrastructure (CNKI), Wanfang Database, China Science Journal Database (VIP), and China Biomedical Database (CBM), was conducted to ensure the thoroughness and accuracy of our research retrieval. Additionally, we searched the Chinese Clinical Trial Registration Center. The search was carried out from the establishment of the databases until November 30, 2023. To ensure an effective search, we employed a combination of theme words and free words, including “astragalus”, “astragalus injection”, “heart failure”, and “ventricular remodeling”. Throughout the search process, we strictly followed the PRISMA guidelines to guarantee comprehensive and accurate retrieval of research articles. Table 1 presents the search strategy used for PubMed, while detailed search strategies for other databases can be found in the Supplementary File S1.

**Table 1:** The search strategy used for pubMed.

No.	Search terms
#1	Astragalus[Title/Abstract]
#2	Astragalus injection[Title/Abstract]
#3	#1 or #2
#4	Heart failure[Title/Abstract]
#5	Ventricular remodeling[Title/Abstract]
#6	#4 or #5
#7	#3 and #6

### 2.3 Type of studies

Inclusion criteria: (a) Study types: Randomized controlled trials (RCTs) published in English or Chinese. (b) Participants: Only patients diagnosed with HFmrEF (NYHA: II-IV), with a LVEF ranging from 41% to 49%. Patients with other types of HF (e.g., heart failure with preserved ejection fraction [HFpEF] or heart failure with reduced ejection fraction [HFrEF]) were excluded. (c) Interventions: The intervention group could include AI as a monotherapy or in combination with standard HF conventional treatment. The control group must receive either a placebo or standard HF conventional treatment. (d) Primary outcomes: Primary outcomes considered were LVEF, left ventricular end-diastolic volume (LVEDV), left ventricular end-diastolic diameter (LVEDD), left ventricular end-systolic volume (LVESV), and left ventricular end-systolic diameter (LVESD). (e) Secondary outcomes: Secondary outcomes included clinical efficacy and N-terminal pro-brain natriuretic peptide (NT-pro BNP) levels.

Exclusion criteria: (a) Non-RCTs. (b) Patients with unstable HF, with a LVEF < 40%. (c) Study on unconventional treatments, such as non-placebo control groups or unconfirmed alternative therapies. (d) Studies without primary outcomes. (e) Only the most comprehensive data from repeated published studies were selected. (f) Studies that could not be accessed online or through email.

### 2.4 Data extraction and quality assessment

In accordance with the inclusion and exclusion criteria, the title and abstract of the included studies were independently screened and retrieved by two reviewers (XH and LH). After excluding obviously unrelated studies, the full-texts of the remaining studies were thoroughly read to determine whether they met the criteria for inclusion. Subsequently, two other reviewers (GL and XM) independently extracted the data from the included studies, including the basic characteristics of the included studies and all outcome indicators.

To assess the risk of bias of the included studies, two reviewers (XH and LH) independently utilized the Cochrane Handbook (18). This tool allowed for the evaluation of potential biases, with assessments categorized as low, high, or unknown risk. To evaluate the quality of the study, the Modified Jadad Scale is used, which encompasses four aspects: random sequence production, allocation concealment, blinding method, withdrawal and dropout, with respective scores of 2, 2, 2, and 1. This scoring system categorizes the quality of RCTs: trials scoring between 1 and 3 are considered of low quality, whereas those scoring between 4 and 7 are deemed high quality. In the event of any discrepancies, the third reviewer (CC) was invited to participate in the discussion to resolve them.

### 2.5 Data analysis

Stata 17.0 software was employed for meta-analysis. For dichotomous data, we expressed the results as relative risk (RR) with 95% confidence interval (CI), while continuous data were presented as mean differences (MD) with 95% CI. *I*^2^ was utilized to assess heterogeneity between included studies. A fixed-effects model was applied when the heterogeneity between studies was minimal (*p* > 0.05, *I*^2 ^< 50%). Conversely, a random-effects model was conducted. We also conducted sensitivity and subgroup analyses to identify potential sources of heterogeneity, along with Begg's and Egger's tests for publication bias assessment.

## 3 Results

### 3.1 Study identification

**Figure 1:**
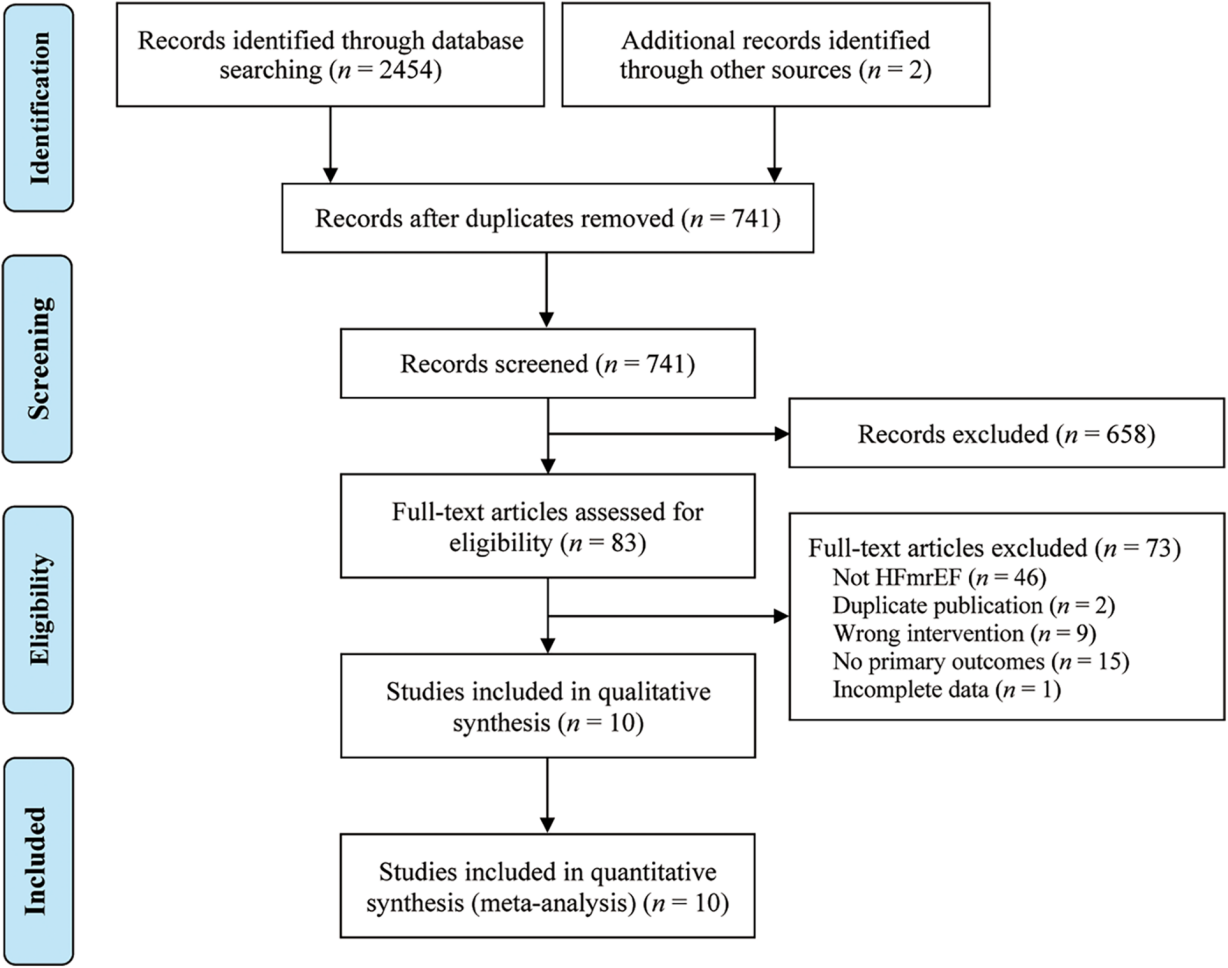
Study flow diagram.

A systematic search was conducted on multiple databases, including PubMed (*n* = 58), Embase (*n* = 43), Cochrane Library (*n* = 30), Web of Science (*n* = 45), CNKI (*n* = 559), Wanfang Data (*n* = 532), VIP (*n* = 650), CBM (*n* = 537), and the Chinese Clinical Trial Registration Center (*n* = 2), retrieving 2,456 potential related original studies. Duplicate studies (*n* = 1,715) were then excluded using Endnote 20.5 software. After reviewing the titles and abstracts, 658 studies were further eliminated. The full texts of 83 studies were read, and ultimately, 73 studies were excluded based on specific criteria. Finally, 10 original studies (19–28) were included in the study. The research selection process is shown in Figure 1.

### 3.2 Included study characteristics

Table 2 presents the basic characteristics of the 10 included studies. A total of 995 patients (562 males and 433 females) were identified, with sample sizes ranging from 30 to 80. The duration of treatment varied from 2 weeks to 4 weeks. The control group received conventional treatment (CT) for HFmrEF according to the HF treatment guidelines. The intervention group received AI combined with CT. The included studies provided the following results: LVEF (19–28), LVEDV (19, 21, 23, 27), LVEDD (20, 24–26, 28), LVESV (19, 21, 23, 27), LVESD (20, 24–26, 28), clinical efficacy (19–28), and NT-pro BNP (19, 20, 22, 24, 25). Among these 10 studies, only 3 studies (19, 25, 27) reported adverse events.

**Table 2:** Included studies basic characteristics.

Study ID	Sample size		NYHA classification						Mean age (years)		Sex (M/F)		Interventions		Treatment duration	Jadad scores	Outcomes
	T	C	T			C			T	C	T	C	T	C			
Chang et al. (19)	47	47		31	16		32	15	67.42 ± 4.92	66.37 ± 5.14	27/20	25/22	AI, 60 ml + CT	CT	4W	4	①②④⑥⑦⑧
Gu et al. (28)	68	66	10	28	30	8	25	33	55.8 ± 6.7		70/64		AI, 40 ml + CT	CT	4W	3	①③⑤⑥
Liu et al. (27)	30	30	6	15	9	7	16	7	68.7 ± 7.2	67.1 ± 7.5	20/10	21/9	AI, 30 ml + CT	CT	2W	4	①②④⑥⑧
Pei (21)	38	38	II–IV						70.23 ± 6.56	69.42 ± 7.15	25/13	26/12	AI, 20 ml + CT	CT	4W	4	①②④⑥
Sun (24)	54	54	II–IV						68.0 ± 8.9	67.0 ± 8.8	25/29	27/27	AI, 40ml + CT	CT	2W	4	①③⑤⑥⑦
Yan and Lin (26)	80	79	II–IV						58.1 ± 11.9	57.8 ± 10.2	54/26	41/38	AI, 50 ml + CT	CT	2W	3	①③⑤⑥
Zhang (20)	59	58	7	32	20	6	31	21	57.79 ± 7.67	58.36 ± 7.34	32/27	30/28	AI, 20 ml + CT	CT	2W	4	①③⑤⑥⑦
Zhang (23)	45	45	15	17	13	14	18	13	56.72 ± 7.45	58.19 ± 7.86	27/18	25/20	AI, 20 ml + CT	CT	2W	4	①②④⑥
Zhao and Zhang (25)	51	46	8	34	9	6	32	8	71.5 ± 8.7		27/24	26/20	AI, 40 ml + CT	CT	2W	4	①③⑤⑥⑦⑧
Zhao et al. (22)	30	30	II–IV						65.5 ± 7.1		34/26		AI, 40 ml + CT	CT	2W	4	①⑥⑦

C, control group; T, treatment group; M, male; F, female; W, weeks; AI, astragalus injection; CT, conventional treatment; NYHA, New York Heart Association. Outcomes: ①LVEF; ②LVEDV; ③LVEDD; ④LVESV; ⑤LVESD; ⑥Clinical efficacy; ⑦NT-pro BNP; ⑧Adverse events.

### 3.3 Risk of bias assessment

**Figure 2:**
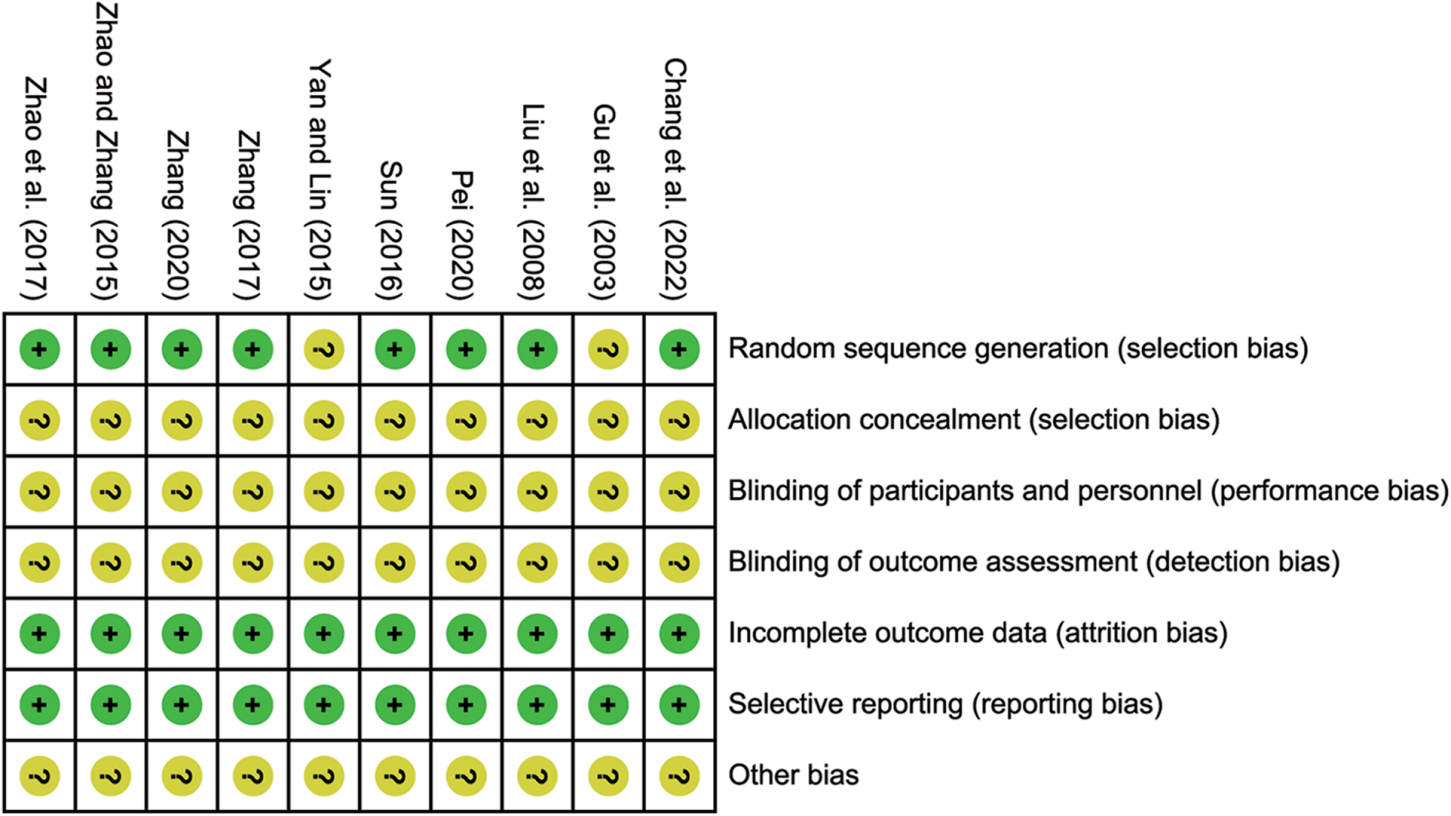
Bias risk assessment of included studies.

The Cochrane bias risk tool was employed to assess the risk of bias. 8 studies (19–25, 27) reported using a random number table method, which were identified as low risk. However, the remaining studies lacked a clear description of their randomization procedures, leading to unclear risk. None of the included studies reported allocation concealment and blinding, contributing to unclear risk. Nevertheless, in terms of selective reporting of incomplete outcome data, all included studies demonstrated no bias, resulting in low risk. The risk bias assessment and the quality assessment are detailed in Figure 2; Supplementary File S2.

### 3.4 Primary outcomes

#### 3.4.1 LVEF

**Figure 3:**
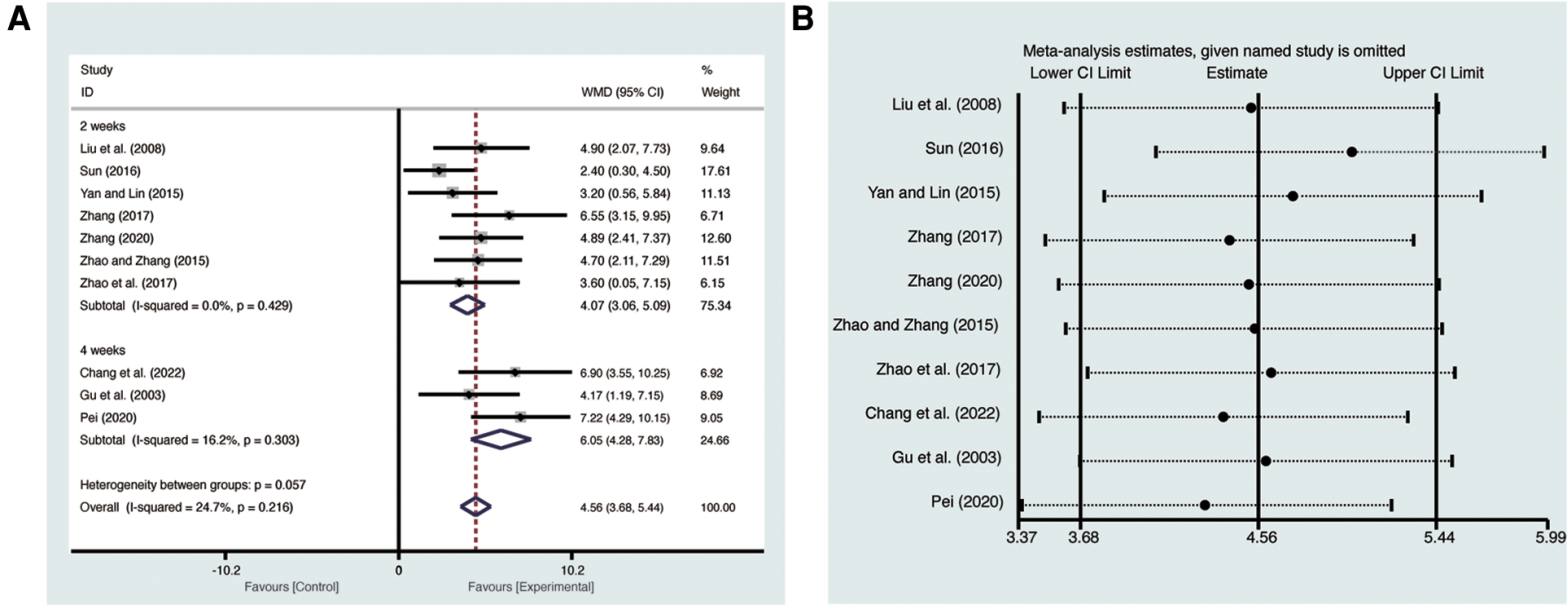
Forest plots with: (**A**) LVEF; (**B**) sensitivity analysis for LVEF.

Ten studies (19–28) evaluated LVEF with low heterogeneity (*I*^2^ = 24.7%, *p* = 0.216) and were merged with a fixed-effects model. Results indicated that AI significantly improved LVEF (MD = 4.56, 95% CI: 3.68–5.44, *p* < 0.00001, Figure 3A). Subgroup analysis based on treatment duration of AI demonstrated significant distinctions between 2 weeks of AI (MD = 4.07, 95% CI: 3.06–5.09, *p *< 0.00001, Figure 3A), 4 weeks of AI (MD = 6.05, 95% CI: 4.28–7.83, *p *< 0.00001, Figure 3A), and CT. Sensitivity analysis confirmed the robustness and reliability of the merged results (Figure 3B).

#### 3.4.2 LVEDV

**Figure 4:**
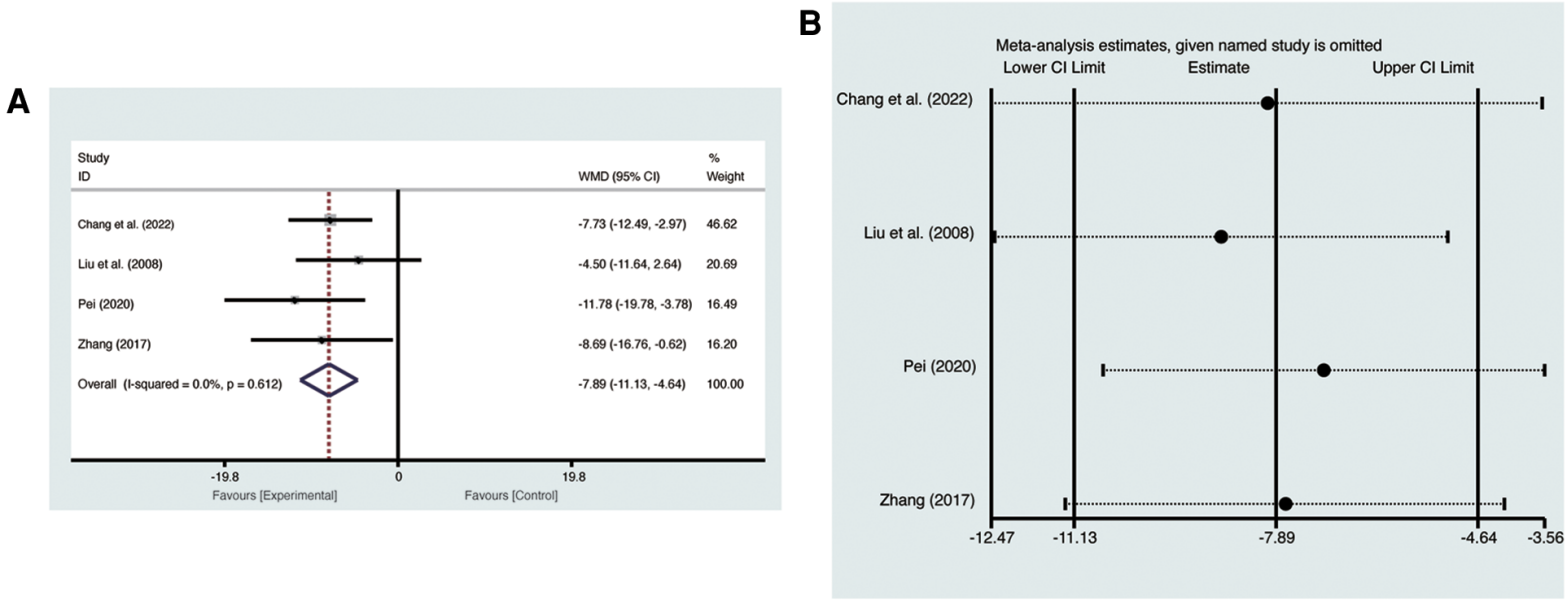
Forest plots with: (**A**) LVEDV; (**B**) sensitivity analysis for LVEDV.

Four studies (19, 21, 23, 27) evaluated LVEDV with low heterogeneity (*I*^2^ = 0%, *p* = 0.612) and were merged with a fixed-effects model. Results indicated that AI significantly reduced LVEDV (MD = −7.89, 95% CI: −11.13 to −4.64, *p* < 0.00001, Figure 4A). Sensitivity analysis confirmed the robustness and reliability of the merged results (Figure 4B).

#### 3.4.3 LVEDD

**Figure 5:**
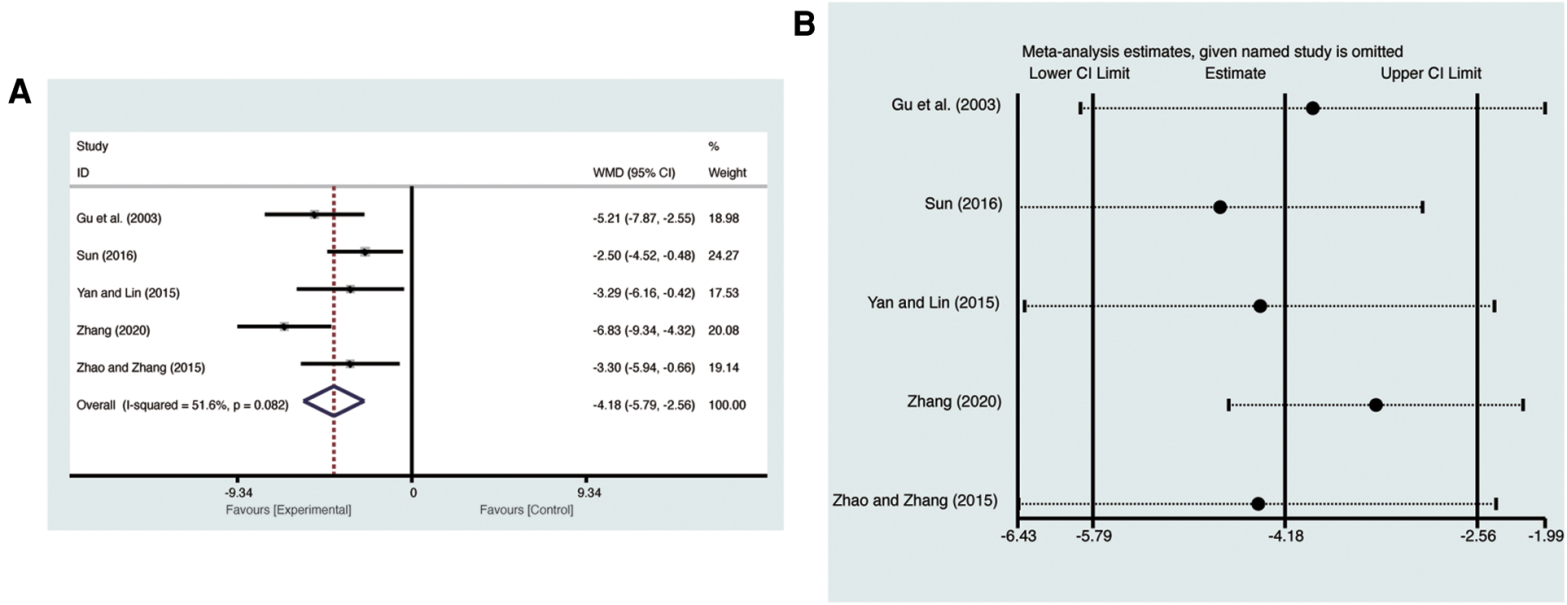
Forest plots with: (**A**) LVEDD; (**B**) sensitivity analysis for LVEDD.

Five studies (20, 24–26, 28) evaluated LVEDD with high heterogeneity (*I*^2^ = 51.6%, *p* = 0.082) and were merged with a random-effects model. Results indicated that AI significantly reduced LVEDD (MD = −4.18, 95% CI: −5.79 to −2.56, *p* < 0.00001, Figure 5A). Sensitivity analysis confirmed the robustness and reliability of the merged results (Figure 5B).

#### 3.4.4 LVESV

**Figure 6:**
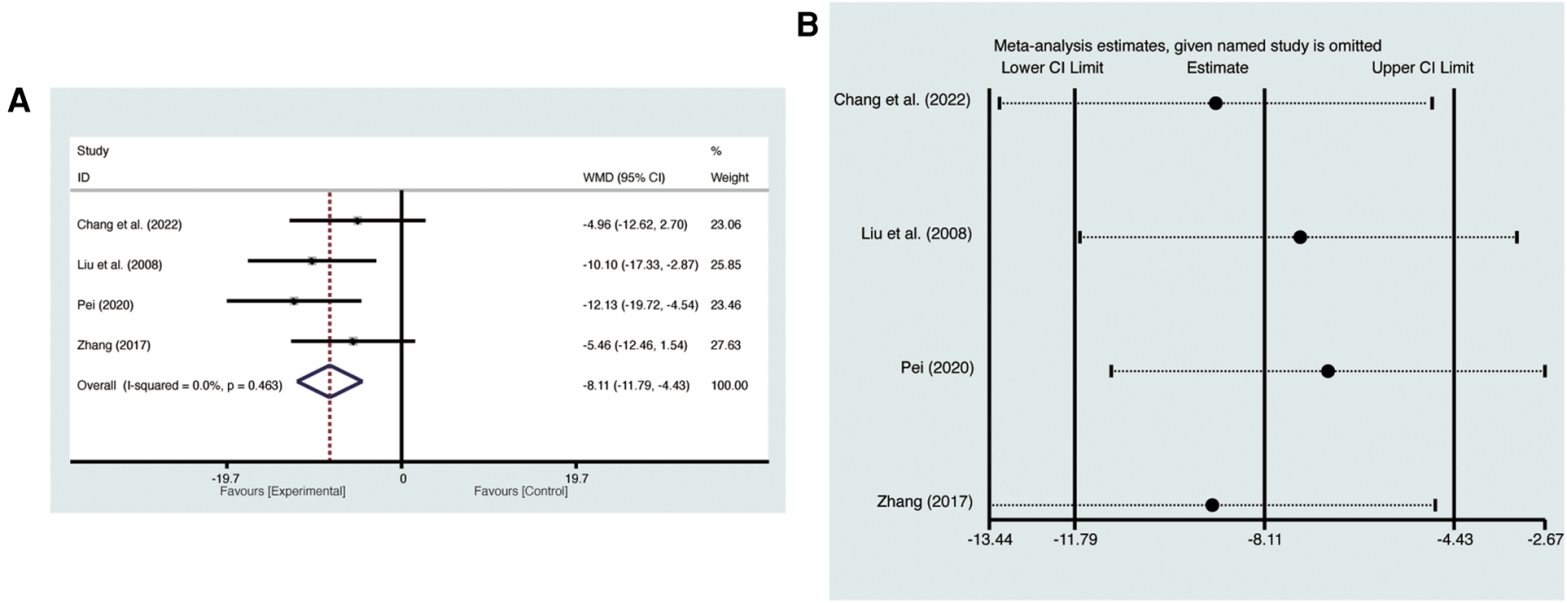
Forest plots with: (**A**) LVESV; (**B**) sensitivity analysis for LVESV.

Four studies (19, 21, 23, 27) evaluated LVESV with low heterogeneity (*I*^2^ = 0%, *p* = 0.463) and were merged with a fixed-effects model. Results indicated that AI significantly reduced LVESV (MD = −8.11, 95% CI: −11.79 to −4.43, *p* < 0.00001, Figure 6A). Sensitivity analysis confirmed the robustness and reliability of the merged results (Figure 6B).

#### 3.4.5 LVESD

**Figure 7:**
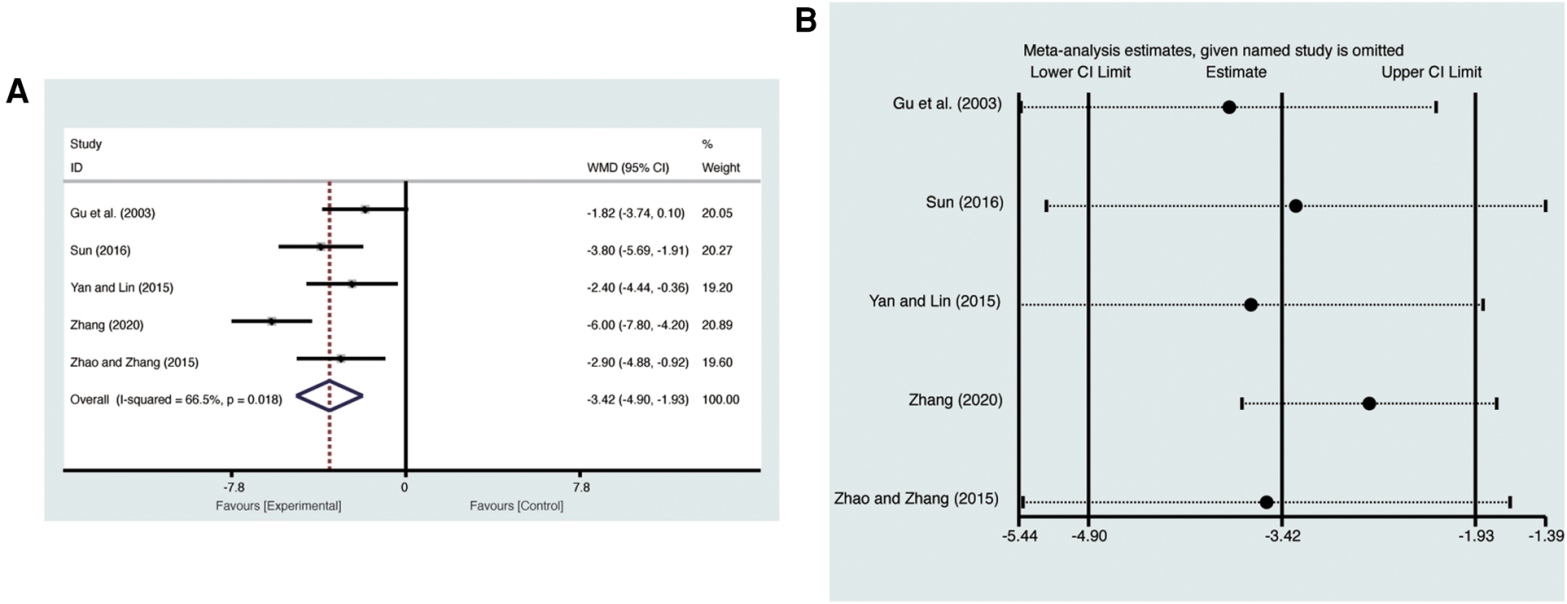
Forest plots with: (**A**) LVESD; (**B**) sensitivity analysis for LVESD.

Five studies (20, 24–26, 28) evaluated LVESD with high heterogeneity (*I*^2^ = 66.5%, *p* = 0.018) and were merged with a random-effects model. Results indicated that AI significantly reduced LVESD (MD = −3.42, 95% CI: −4.90 to −1.93, *p* < 0.00001, Figure 7A). Sensitivity analysis confirmed the robustness and reliability of the merged results (Figure 7B).

### 3.5 Secondary outcomes

#### 3.5.1 Clinical efficacy

**Figure 8:**
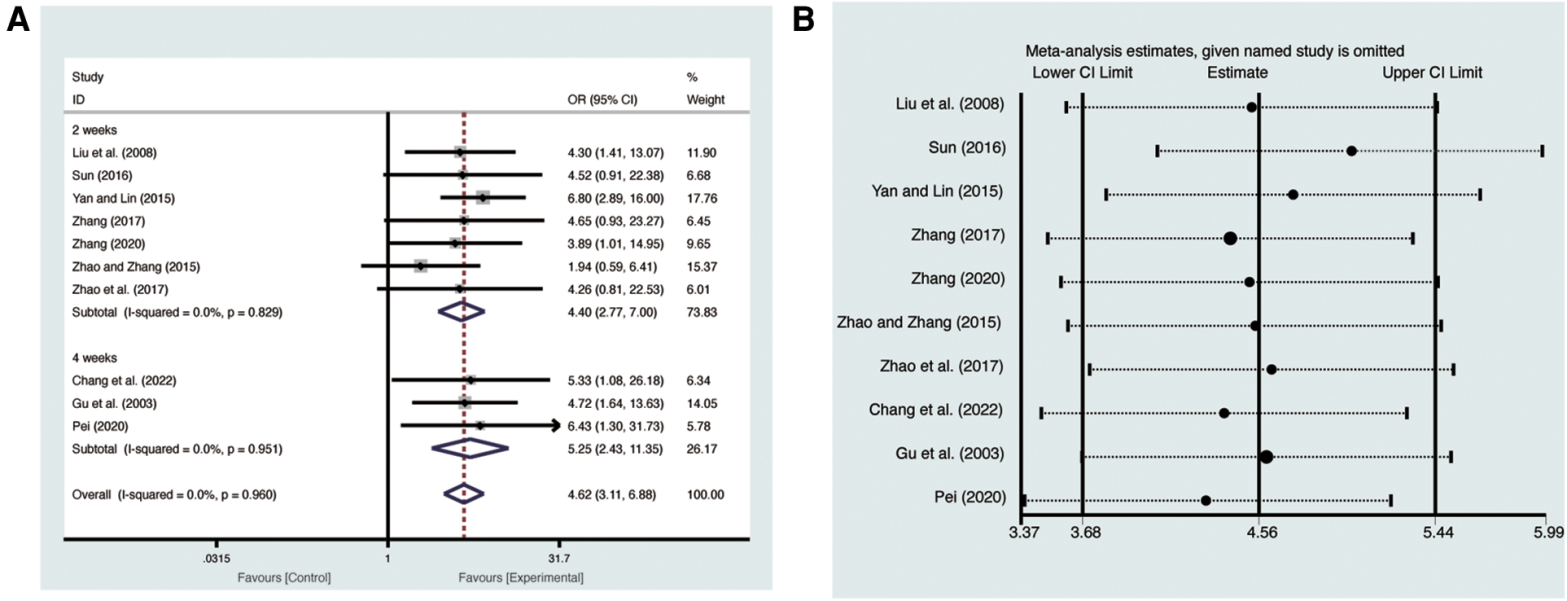
Forest plots with: (**A**) clinical efficacy; (**B**) sensitivity analysis for clinical efficacy.

Ten studies (19–28) evaluated clinical efficacy with low heterogeneity (*I*^2^ = 0%, *p* = 0.960) and were merged with a fixed-effects model. Results indicated that AI significantly improved clinical efficacy (RR = 4.62, 95% CI: 3.11–6.88, *p* < 0.00001, Figure 8A). Subgroup analysis based on treatment duration of AI demonstrated significant distinctions between 2 weeks of AI (RR = 4.40, 95% CI: 2.77–7.00, *p *< 0.00001, Figure 8A), 4 weeks of AI (RR = 5.25, 95% CI: 2.43–11.35, *p *< 0.0001, Figure 8A), and CT. Sensitivity analysis confirmed the robustness and reliability of the merged results (Figure 8B).

#### 3.5.2 NT-pro BNP

**Figure 9:**
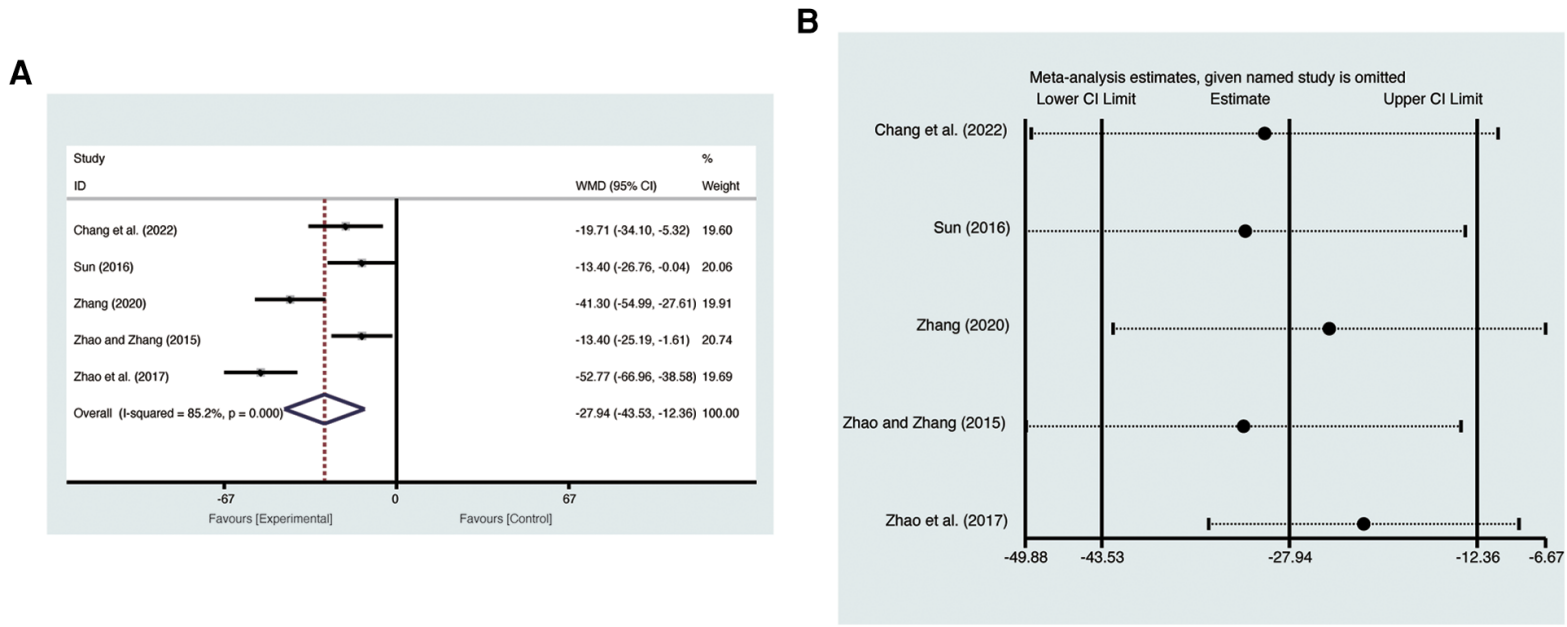
Forest plots with: (**A**) NT-pro BNP; (**B**) sensitivity analysis for NT-pro BNP.

Five studies (19, 20, 22, 24, 25) evaluated NT-pro BNP with high heterogeneity (*I*^2^ = 85.2%, *p* = 0.000) and were merged with a random-effects model. Results indicated that AI significantly reduced NT-pro BNP (MD = −27.94, 95% CI: −43.3 to −12.36, *p* = 0.0004, Figure 9A). Sensitivity analysis confirmed the robustness and reliability of the merged results (Figure 9B).

#### 3.5.3 Adverse events

Only 3 (19, 25, 27) of the 10 included studies reported adverse events. These adverse events included dizziness, headache, palpitations, nausea, and elevated transaminases. However, the merged results indicated that there was no significant difference in adverse events between the two groups (RR = 1.60, 95% CI: 0.59–4.29, *p* = 0.35). Table 3 provided detailed adverse events.

**Table 3:** The incidence rate of adverse events.

Adverse events symptoms	Study ID	The number of adverse events	
		T	C
Dizziness	Chang et al. (19); Liu et al. (27)	3	2
Headache	Zhao and Zhang (25)	3	1
Palpitations	Liu et al. (27)	1	1
Nausea	Chang et al. (19)	1	1
Elevated transaminases	Zhao and Zhang (25)	2	1
Total reactions	–	10/128	6/123
Incidence rate	–	7.81%	4.89%

### 3.6 Publication bias

**Figure 10:**
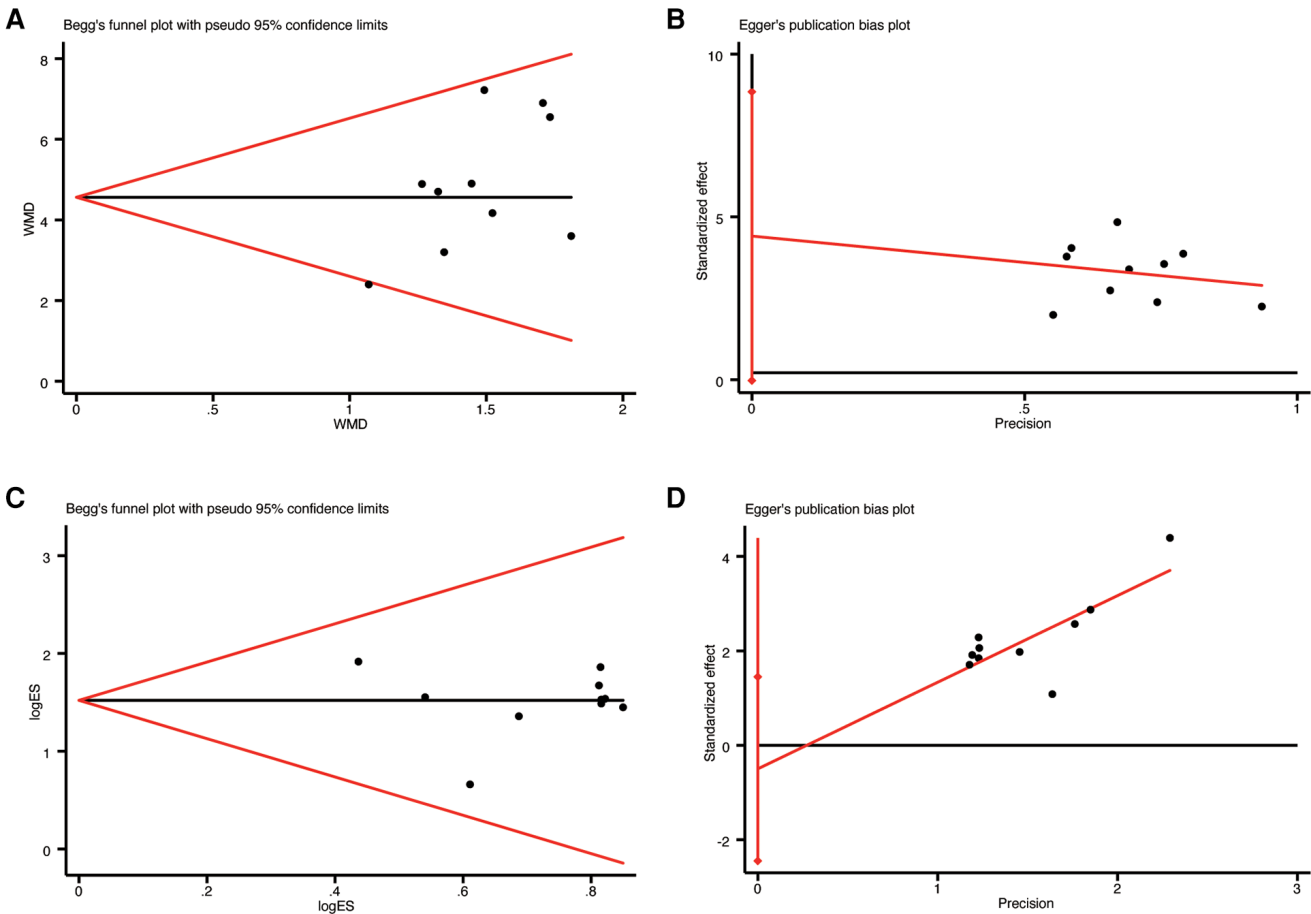
The results of publication bias: (**A**) begg's funnel plot of LVEF; (**B**) egger's funnel plot of LVEF; (**C**) begg's funnel plot of clinical efficacy; (**D**) egger's funnel plot of clinical efficac.

Ten studies (19–28) that reported LVEF and clinical efficacy were evaluated for publication bias using Begg's and Egger's tests.The results of the Begg's test indicated no significant publication bias for LVEF (*p* = 0.474, Figure 10A) and clinical efficacy (*p* = 0.721, Figure 10C). Similarly, the Egger's test showed no significant publication bias between LVEF (*p* = 0.051, Figure 10B) and clinical efficacy (*p* = 0.571, Figure 10D).

## 4 Discussion

HFmrEF, defined in the 2021 ESC guidelines as a subtype of HF, is characterized by left ventricular remodeling (LVR), which includes changes in ejection fraction and ventricular volumes (3). These pathological features have been shown to be associated with increased hospitalization and mortality risk, underscoring the need for effective treatment strategies (29). However, the majority of research on HF has focused on heart failure with reduced ejection fraction (HFrEF), with less attention given to HFmrEF (30, 31). While the 2021 ESC guidelines suggest that angiotensin converting enzyme (ACE) inhibitors, angiotensin receptor blockers (ARBs), diuretics, and β receptor blockers may have potential benefits for HFmrEF patients, the evidence supporting their effectiveness is relatively low (3). In light of this, there is an urgent need to explore alternative and complementary therapies to improve LVR in patients with HFmrEF.

*Astragalus membranaceus* is a medicinal and edible homologous species in China with a long history of application in food and clinical practice, commonly used to treat HF. AI mainly consists of extracts from *Astragalus membranaceus*, containing various active components such as saponins, polysaccharides, and flavonoids. These components exert anti-HF effects through multiple mechanisms, including anti-inflammatory, antioxidant, anti-fibrotic, regulation of cellular hypertrophy, and inhibition of senescence (32–34). Astragaloside IV (AS-IV) alleviates HF by inhibiting CCL2-mediated NF-κB signaling pathway activation, reducing LPS-induced myocardial hypertrophy and collagen deposition (35). Additionally, AS-IV mitigates ISO-induced myocardial fibrosis by inhibiting oxidative stress and regulating the P53 signaling pathway and cellular senescence (36). Astragalus polysaccharides (APS) reduce ISO-induced myocardial hypertrophy by regulating energy biogenesis mediated by the TNF-α/PGC-1α signaling pathway (37). Furthermore, APS improve doxorubicin-induced cardiotoxicity by regulating the PI3k/Akt and p38MAPK pathways to inhibit oxidative stress and apoptosis (38). However, despite multiple studies confirming its effectiveness in treating HFmrEF, there is a lack of evaluation regarding its impact on LVR. Since LVR plays a crucial role in the pathogenesis and progression of HFmrEF, this article aims to conduct a meta-analysis of randomized controlled trials (RCTs) to systematically evaluate the influence of AI on LVR in HFmrEF patients. The findings of this analysis aim to provide more reliable evidence for clinical decision-making in the treatment of HFmrEF.

### 4.1 Summary of findings

This meta-analysis is the first to investigate the effect of AI on LVR in patients with HFmrEF. The following findings were observed: (a) Compared to CT alone, the combination of AI and CT resulted in increased LVEF and decreased LVEDV, LVEDD, LVESV, LVESD, along with reduced NT-pro BNP levels. (b) Additionally, compared to CT alone, the combination of AI and CT exhibited increased clinical efficacy. (c) Importantly, the combination of AI and CT did not lead to an increase in adverse events. Based on these results, our meta-analysis suggests that AI may effectively improve LVR in patients with HFmrEF and enhance overall clinical efficacy.

The sensitivity analysis, which involved sequentially deleting individual studies, confirmed the robustness and reliability of the merged results. Additionally, to evaluate publication bias, STATA 17.0 software was used to conduct Begg's and Egg's tests. The analysis revealed no significant bias in the included studies.

### 4.2 Strengths and limitations

Currently, some researchers have conducted meta-analysis on the treatment of HF with AI (39). However, these studies have not considered the clinical heterogeneity among HF patients with different ejection fractions. It has been demonstrated that there may be significant differences in the pathological and physiological processes of HF with different ejection fraction types (40). Therefore, we believe that studying different types of HF is more meaningful for exploring personalized treatment plans. Furthermore, previous studies have focused primarily on the clinical efficacy and adverse reactions of AI, neglecting the crucial role of LVR in the progression of HF. In contrast, our study went beyond these limitations and evaluated the effect of AI on LVR in HFmrEF patients.

Additionally, there are several limitations to our study. First, the overall quality of the RCTs included is relatively low. Some of the included studies did not report randomized methods, blinding, and allocation concealment, which may lead to a serious risk of bias. Second, some of the RCTs included in this study are relatively small in scale, and therefore, the results should be interpreted with caution. Third, there are only three studies that reported adverse events; further investigation is needed to ensure the safety of AI. Fourth, none of the included studies reported follow-up time, indicating the necessity for further research to investigate the long-term effects of AI. Fifth, the study exclusively focused on patients with HFmrEF, thus excluding those with HFpEF and HFrEF. Therefore, the results may not be generalizable to all HF patients. Lastly, some of our results exhibit moderate to high heterogeneity, and conducting sensitivity analysis alone is not sufficient to draw clear conclusions. Therefore, further research is necessary to verify the reliability of our research findings.

### 4.3 Implication

In order to enhance the evidence strength of AI in treating HFmrEF, future clinical research should focus on the following aspects. Firstly, HF should be classified according to guidelines to reduce sources of bias and draw more accurate conclusions. Secondly, more high-quality RCTs should be conducted with a subject-centered approach, implementing strict randomization, allocation concealment, and blinding techniques. Thirdly, it is crucial to use a comprehensive standard reporting trial statement to report RCTs in a complete and comprehensive manner (41). This should include reporting medical history, readmission rate, quality of life, and follow-up time to analyze sources of heterogeneity and clarify the prognosis of HFmrEF. Fourthly, the included studies have relatively small sample sizes, future research should conduct large-scale, multicenter, prospective clinical trials and extend them to other populations to fully evaluate the effects of AI on HFmrEF. Fifthly, future research should also investigate the effects of AI on patients with HFpEF and HFrEF, which will help to understand the potential benefits and safety of AI across different HF populations. By addressing these aspects, future research can provide stronger evidence for the effectiveness of AI in treating HFmrEF, as well as HFpEF and HFrEF.

## 5 Conclusion

The systematic review and meta-analysis results indicate that combining AI with CT improves LVR in HFmrEF patients. This improvement is evidenced by increased LVEF, reduced LVEDV, LVEDD, LVESV, and LVESD, along with improved clinical efficacy and reduced NT-pro BNP levels without increasing adverse events. However, caution is needed in interpreting the results due to the low level of evidence. Future high-quality RCTs are needed to support these conclusions.

## Data Availability

The original contributions presented in the study are included in the article/Supplementary Material, further inquiries can be directed to the corresponding author.
