# Supplementary material for: Effect of astragalus injection on left ventricular remodeling in HFmrEF: a systematic review and meta-analysis
Source: Front Cardiovasc Med. 2024 Aug 6;11:1374114. doi: 10.3389/fcvm.2024.1374114 (PMC11333324; doi:10.3389/fcvm.2024.1374114)
Supplement: Supplementary file 1 [file Table1.docx]

Supplementary Material

**Effect of astragalus injection on left ventricular remodeling in HFmrEF: a systematic review and meta-analysis**

Xu Han^1^, Lumei Huang^2^, Geng Li^2^, Xinglang Mou^2^ and Caihong Cheng ^2^*

*^1^ Chongqing Changshou Traditional Chinese Medicine Hospital, Chongqing, China, ^2^ Traditional* *Chinese Medicine Hospital Dianjiang Chongqing, Chongqing, China*

**Correspondence: Caihong Cheng, 873979603@qq.com. Traditional Chinese Medicine Hospital Dianjiang Chongqing, Chongqing, 408300, China.*

**Supplementary** **File S1. Search strategies.**

**Search run on November 30, 2023**

**PubMed (*n*=58)**

#1 ((astragalus[Title/Abstract]) OR (astragalus injection[Title/Abstract]))

#2 ((heart failure[Title/Abstract]) OR (ventricular remodeling[Title/Abstract]))

#3 #1 AND #2

**Embase (*n*=43)**

#1 'astragalus'/exp

#2 'astragalus injection'/exp

#3 #1 OR #2

#4 'heart failure'/exp

#5 'ventricular remodeling'/exp

#6 #4 OR #5

#7 #4 AND #5

**Cochrane library (*n*=30)**

#1 MeSH descriptor: [astragalus] explode all trees

#2 astragalus injection*.ti,ab,kw

#3 #1 or #2

#4 MeSH descriptor: [heart failure] explode all trees

#5 ventricular remodeling *.ti,ab,kw

#6 #4 or #5

#7 #3 and #6

**Web of Science (*n*=45)**

#1 (TS=(astragalus) OR ALL=( astragalus injection))

#2 (TS=(heart failure) OR ALL=(ventricular remodeling))

#3 #1 AND #2

**CNKI (*n*=559)**

#1 篇关摘：黄芪注射液

#2 篇关摘：心力衰竭 OR 心室重构

#3 #1 AND #2

**Wanfang Data (*n*=532)**

#1 主题：黄芪注射液

#2 主题：心力衰竭 OR 心室重构

#3 #1 AND #2

**VIP (*n*=650)**

#1 篇关摘：黄芪注射液

#2 篇关摘：心力衰竭 OR 心室重构

#3 #1 AND #2

**CBM (*n*=537)**

#1 常用字段：黄芪注射液

#2 常用字段：心力衰竭 OR 心室重构

#3 #1 AND #2

**中国临床试验注册中心 (*n*=2)**

**Supplementary File S2. Modified Jadad Scores for 10 studies.**

| **Study ID** | **Random sequence production** | **Allocation concealment** | **Blind method** | **Withdrawal and Dropouts** | **Total scores** |
| --- | --- | --- | --- | --- | --- |
| Chang et al. (2022) | 2 | 1 | 0 | 1 | 4 |
| Gu et al. (2003) | 1 | 1 | 0 | 1 | 3 |
| Liu et al. (2008) | 2 | 1 | 0 | 1 | 4 |
| Pei (2020) | 2 | 1 | 0 | 1 | 4 |
| Sun (2016) | 2 | 1 | 0 | 1 | 4 |
| Yan and Lin (2015) | 1 | 1 | 0 | 1 | 3 |
| Zhang (2017) | 2 | 1 | 0 | 1 | 4 |
| Zhang (2020) | 2 | 1 | 0 | 1 | 4 |
| Zhao and Zhang (2015) | 2 | 1 | 0 | 1 | 4 |
| Zhao et al. (2017) | 2 | 1 | 0 | 1 | 4 |
